# Age-group-specific association of oral health and systemic health on cognitive function: a cross-sectional study of Korean elders

**DOI:** 10.1186/s12903-023-03724-2

**Published:** 2023-12-13

**Authors:** Jae-Eun Sin, Hye-Sung Kim, Inseong Hwang, Miwha Noh

**Affiliations:** 1 Apple Tree Institute of Biomedical Science, Apple Tree Medical Foundation, 1450 Jungang-ro, Goyang-si, Gyeonggi-do Republic of Korea; 2 Apple Tree Dental Hospital, Apple Tree Medical Foundation, 1450 Jungang-ro, Goyang-si, Gyeonggi-do Republic of Korea; 3 DOCSmedi OralBiome Co., Ltd, 143 Gangseong-ro, Goyang-si, Gyeonggi-do Republic of Korea

**Keywords:** Age group, Cognitive function, Elders, Korean Longitudinal Study of Aging, Oral health, Systemic health

## Abstract

**Background:**

Although the importance of oral and systemic healthcare for elderly people is increasing owing to the rapid ageing of the population in South Korea, studies on the relationship between oral health, systemic health, and cognitive function, as well as on the prediction of cognitive function by oral and systemic health depending upon age groups are lacking.

**Methods:**

We included 5,975 out of 6,488 participants from the 8^th^ wave of the Korean Longitudinal Study of Aging (KLoSA) panel data, divided the participants into three age groups, and performed a hierarchical multiple linear regression analysis to explain cognitive function with four types of predictors: oral health status, sociodemographic factors, objective health status, and subjective health status.

**Results:**

Oral health status was positively correlated with systemic health status and cognitive function. Of all ages over 54, cognitive function was significantly predicted by oral health variables, such as the number of functional teeth, masticatory ability, and Geriatric Oral Health Assessment Index (GOHAI); sociodemographic variables, such as age, sex, education level, and residence; and systemic health variables, such as diagnosis of diabetes mellitus, cancer or malignant tumours, cerebrovascular disease and rheumatoid arthritis, depressive symptom, and self-rated health status. Oral health variables explained cognitive function differently by age group; GOHAI appeared important predictor in the group aged < 75 years, whereas the number of functional teeth did in the group aged ≥ 75 years. Educational level, masticatory ability, depressive symptoms, and self-rated health status were pivotal factors age-independently.

**Conclusions:**

The general and age-group-specific association between oral health, systemic health, and cognitive function were confirmed, suggesting that age-group-specific oral healthcare should be emphasized for the effective management of systemic and cognitive health in the elderly group.

**Supplementary Information:**

The online version contains supplementary material available at 10.1186/s12903-023-03724-2.

## Background

By 2050, the share of the global proportion aged > 65 years will reach 16% [1]. South Korea is expected to become a super-aged society by 2025 when the proportion of the elderly aged 65 years or above becomes 20.6% from 17.5% in 2022 [2]. In 2020, 840,000 (10.3%) of the elderly population in Korea were diagnosed with dementia, which will rise to three million (16%) by 2050 [3]. The national cost for the management of dementia in South Korea is expected to reach $65 billion in 2050 from $12.7 billion in 2020 [3]. In the absence of a clear cure for dementia, diagnosis of an early stage of the disease, e.g., mild cognitive impairment (MCI), followed by holistic healthcare to delay the onset of the disease is accepted as the best practice.

Growing evidence emphasizes the link between oral health and cognitive function in elderly people [4–6]. Indeed, many of the risk factors of systemic diseases, such as cardiovascular and circulatory diseases [7–9], rheumatoid arthritis [10, 11], and Alzheimer’s disease [12], coincide with the ones of oral diseases [13, 14]. Among the variables for oral health, periodontitis [15–17], the number of teeth [18–20], chewing efficiency [21–23], and infection by a complex set of bacterial species [12, 24, 25] have been reported to be correlated with cognitive impairment. Furthermore, several clinical studies have shown that oral rehabilitation [22, 26] and hygienic interventions [27, 28] may be effective in improving cognitive function. Recently, Asher and colleagues conducted a systematic review and meta-analysis of 47 longitudinal studies, published until April 2022, finding that poor periodontal health and tooth loss appear to increase the risk of cognitive impairment [29]. In addition, they also found, albeit with limited evidence, that the extent of tooth loss is proportional to the risk of cognitive impairment; partial tooth loss is important for cognitive decline whereas complete tooth loss is for dementia.

The Korean Longitudinal Study of Aging (KLoSA), launched in 2006, is a biannual nationwide panel survey of people aged 45 and older and their partners, focusing on international data compatibility, cross-disciplinary studies, and socio-economic policy making [30]. When the 7th wave of KLoSA was conducted, oral health-related questionnaires were newly adopted as the growing number of studies had enunciated the importance of oral health in late-life satisfaction with respect to the linkage between oral health and cognitive ability. Of particular, studies using KLoSA have identified irregularity in working hours [31], low life satisfaction [32], decreased chewing function [33], and the risk factors of cardiovascular diseases (CVD), such as age, diabetes, and hypertension [34], are associated with the development of cognitive impairment. It was also reported that depression and cognitive function are associated with oral health-related quality of life in elderly people aged 65 years and over using the 7th wave of KLoSA data [35]. However, a scarce number of studies exploring the association among oral health, systemic health, sociodemographic factors, as well as cognitive function altogether in elderly people have been introduced yet.

Traditionally, 65 years of age has been recognised as the start of the older age eligible for the official retirement based on social and economic feasibility, rather than biological definition [36]. Although age classification for elderly people varies between countries, efforts to re-define the elderly as aged 75 years or over have emerged especially in East Asian countries with increased life expectancy and rapid population ageing [37]. However, no studies have been reported to explain cognitive function by oral health status, systemic health status, and sociodemographic characteristics in different age groups in Korean older adults. Thus, this study, in pursuit of the traditional definition of elders in Korea [38, 39], aimed to identify the different associations between oral or systemic health status and cognitive function in Korean elders by three age groups (the pre-, the early-, and the middle-or-late-elderly), taking into consideration on the age distribution of the 8^th^ KLoSA dataset.

## Methods

### Data collection and study design

In this study, sample data were collected from the 8^th^ wave of the KLoSA issued by the Korea Employment Information Service (KEIS) in 2020. The first wave of the survey comprised 4,460 men and 5,794 women out of 10,254, randomly selected people aged 45 and over (born before 1961) by stratified multistage probability sampling from nationwide, excluding Jeju Island in 2006 (baseline). In the 5^th^ wave (2014), a sample of 920 people born between 1962 and 1963 was added [30]. So far, 6,488 people have completed the survey every two years until 2020 (8^th^ wave).

The survey was conducted by computer-assisted personal interviewing (CAPI) using a structured questionnaire assessing household background, socio-demographics, family structure, healthcare, employment status, income, assets, and subjective life satisfaction. All interviewers and investigators recruited were not professional medical practitioners, so they had to be managed, and pre-trained to abide by data collection protocols or guidelines provided by KEIS every two years.

Sample data for our cross-sectional secondary analysis study using the 8^th^ wave of the KLoSA were provided as three types, raw data, structurally converted data, and light version data. The KLoSA survey were reviewed and approved by the Institutional Review Board of Statistics Korea (approval number: 336,052) and this secondary analysis study, using the KLoSA data, was also conducted after approval from the Public Institutional Review Board (http://public.irb.or.kr) by the Korea National Institute for Bioethics Policy (approval number: P01-202206-01-003).

### Study parameters

#### Independent variables

Sociodemographic variables: Sex, age, marital status, educational level, and residence were selected as sociodemographic variables having been reported as denoted factors both associated with oral health status [40, 41] and cognitive impairment [42, 43] in the elderly group. The marital status was classified as “unmarried”, “widowed”, “divorced”, “living apart together”, and “married”. The level of education was divided into “elementary school”, “middle school”, “high school”, and “college” graduates. The residence was classified into “township”, “small city”, and “big city”.

Oral health variables: For oral health, the number of functional teeth, masticatory ability, and the Korean version of GOHAI were used [44] and those variables had all been reported to have bidirectional relation to cognitive function in the literature [45]. Based on data user guidelines by KEIS, responders were asked four items regarding their teeth, which included the number of implant teeth, teeth in dentures, missing teeth to be replaced by implants or dentures, and remaining wisdom teeth. Assuming the basic number of 28 natural teeth, it is stated that the number of natural teeth was counted by summing 28 and the number of remaining wisdom teeth and subtracting the number of missing teeth as well as false teeth, such as implants or dentures. Using responses from four items and the newly created ‘natural teeth’ variable in the sample data, we calculated the number of functional teeth by adding the number of implants to the number of natural teeth and then subtracting the number of wisdom teeth. Masticatory ability was measured using a subjective five-point Likert-type scale (1 = very good chewing, 2 = good chewing, 3 = moderate, 4 = poor chewing, 5 = no chewing at all). The original version of GOHAI, a 12-item measure with a six-point scale scoring system, assesses oral-related pain/discomfort, physical function, and psychosocial function (0 = always, 1 = very often, 2 = often, 3 = occasionally, 4 = rarely, 5 = never). Response from the masticatory ability item was reversely coded in this study.

Systemic health variables: As recent studies have been shedding light on the interaction between oral health and systemic health [46], this study categorized systemic health into objective health status (the number of chronic diseases, the diagnosis of disease, alcohol use habit, and smoking habit) and subjective health status (depressive symptom and self-rated health status) to explore the oral-overall health relationship. For the diagnosis of disease, the responses to individual diagnostic status of ten diseases (hypertension, diabetes mellitus, cancer and malignant tumours, chronic lung disease, liver disease, heart disease, cerebrovascular disease, psychiatric disease, rheumatoid arthritis, and digestive disorders) were dichotomized into “yes” and “no”. The number of chronic diseases was generated as the sum of the number of “yes” responses for the presence of the aforementioned diseases. Alcohol use and smoking habits were classified into “never”, “former”, and “current”. The depressive symptom was measured using the Korean version of the Centre for Epidemiologic Studies Depression Scale (CES-D10) [47]. Self-rated health status by the question ‘How do you evaluate your health status?‘ with a five-point Likert-type scale (1 = very good, 2 = good, 3 = average, 4 = bad, 5 = very bad) was reverse-coded for the analysis.

#### Dependent variables

Cognitive function variables: The Korean version of the Mini-Mental State Examination (K-MMSE) was used to evaluate the cognitive state of the participants [48, 49]. The K-MMSE, consisting of 19 elements, has scores ranging from 0 to 30 points with higher scores indicating better cognitive function.

### Data analysis

In this study, four datasets were prepared for data analysis; one was a total dataset with entire subjects for performing a descriptive analysis, correlation analysis, and hierarchical multiple linear regression modelling based on four conceptual models; three other datasets were the subgroups of the total dataset divided by their ages “under 65”, “between 65 and 74” and “75 and older” that were pre-defined classification of the pre-old, the early-old, and the middle-or-late-old population for conducting descriptive analysis and multiple linear regression analysis [38, 39]. Descriptive statistics were applied to delineate the general characteristics of the study subjects for all variables. To identify the statistically significant age group differences in the values or ratios of discrete and continuous variables, a chi-square test and one-way analysis of variance (ANOVA) were performed, respectively. Welch’s ANOVA was conducted on the variables provided that the homogeneity of variance was not confirmed by Bartlett’s test. With regards to correlation analysis, Pearson’s coefficient was calculated to explore the correlation between continuous variables, otherwise Spearman’s coefficient was computed. A hierarchical multiple linear regression analysis using a total dataset was conducted to explain cognitive function by predictors when building up the model with (a) oral health variables, (b) sociodemographic variables, (c) objective health status variables, and (d) subjective health status variables in sequential order. Additional subgroup analysis results were compared to predict the cognitive function of elderly people by age group while entering all the predictors simultaneously into a multiple linear regression model. All categorical predictors were treated as dummy variables and statistical assumptions were scrutinized before executing regression analysis. Data processing and analysis were implemented in R (version 4.1.0) and the significance level was set to 0.05 for all statistical inferences carried out in the study.

## Results

### General characteristics of study participants

The general characteristics of the 5,975 subjects, excluding missing values of the dependent variables ​​from the total of 6,488 who completed the survey, are shown in < Table 1>. The average age of the subjects was 70.8 years (32.5%, under 64; 32.2%, 65–74; and 35.3%, ≥ 75), which consisted of 3,468 females (58.0%) and 2,507 males (42.0%). The education level was elementary school graduates (36.9%), middle school graduates (16.9%), high school graduates (33.3%), or university graduates (12.9%). In terms of marital status, the majority of people were married (75.0%) or widowed (21.1%), whereas others remained unmarried (0.9%), divorced (2.5%), or living apart together (0.5%). The residence area was classified as big city (42.3%), small city (34.6%), and township (23.1%). It revealed significant associations between age and sociodemographic characteristics (education level, χ^2^(6) = 1553.50, *p* < .001; marital status, χ^2^(8) = 919.54, *p* < .001; area of residence, χ^2^(4) = 107.05, *p* < .001). On average, the number of functional teeth, masticatory ability and the GOHAI score were 22.8 (SD = 9.5), 3.2 (SD = 0.8), and 35.9 (SD = 7.0), respectively, and there showed statistically significant association of age group on oral health (number of functional teeth, *F*(2, 3642) = 637.36, *p* < .001; masticatory ability, *F*(2, 3974) = 665.40, *p* < .001; GOHAI, *F*(2, 3965.8) = 250.98, *p* < .001). Subjects had an average of 1.3 chronic diseases and had been commonly diagnosed with hypertension (46.0%), rheumatoid arthritis (25.6%), diabetes mellitus (21.0%), and heart disease (9.9%). People had experienced more alcohol use than smoking; 51.5% had alcohol drinking experience (20.5% stopped drinking, and 31.0% currently drinking); 30.8% had smoking experience (22.9% quitted smoking, and 7.9% currently smoking). Regarding subjective health status, the average degree of depressive symptoms and self-rated health status were 1.3 (SD = 1.8), and moderate with 3.0 (SD = 0.8), respectively. The average level of cognitive function was 25.1 (SD = 5.4) and significantly different by age group, *F*(2, 3718.5) = 802.36, *p* < .001.

**Table 1:** Table of descriptive statistics

	Total (*n* = 5975)	< 65 (*n* = 1942)	65–74 (*n* = 1923)	≥ 75 (*n* = 2110)	*F*/*χ*^2^
	*N* (%) / *M* (SD)	*N* (%) / *M* (SD)	*N* (%) / *M* (SD)	*N* (%) / *M* (SD)	
Sex					11.13 (2)^**^
male	2507 (42.0)	815 (42.0)	859 (44.7)	833 (39.5)	
female	3468 (58.0)	1127 (58.0)	1064 (55.3)	1277 (60.5)	
Education level					1553.50 (6)^***^
elementary school	2205 (36.9)	194 (10)	630 (32.8)	1381 (65.4)	
middle school	1008 (16.9)	293 (15.1)	449 (23.3)	266 (12.6)	
high school	1990 (33.3)	1028 (52.9)	633 (32.9)	329 (15.6)	
college	772 (12.9)	427 (22.0)	211 (11.0)	134 (6.4)	
Marital status					919.54 (8)^***^
unmarried	51 (0.9)	32 (1.6)	14 (0.7)	5 (0.2)	
widowed	1264 (21.1)	100 (5.1)	279 (14.5)	885 (42.0)	
divorced	148 (2.5)	73 (3.8)	48 (2.5)	27 (1.3)	
living apart together	32 (0.5)	13 (0.7)	14 (0.7)	5 (0.2)	
married	4480 (75.0)	1724 (88.8)	1568 (81.6)	1188 (56.3)	
Area of residence					107.05 (4)^***^
township	1382 (23.1)	312 (16.1)	443 (23.0)	627 (29.7)	
small city	2064 (34.6)	717 (36.9)	668 (34.8)	679 (32.2)	
big city	2529 (42.3)	913 (47.0)	812 (42.2)	804 (38.1)	
Chronic disease					
hypertension	2746 (46.0)	502 (25.8)	902 (46.9)	1342 (63.6)	581.33 (2)^***^
diabetes mellitus	1257 (21.0)	206 (10.6)	428 (22.3)	623 (29.5)	220.42 (2)^***^
cancer or malignant tumours	453 (7.6)	96 (4.9)	162 (8.4)	195 (9.2)	29.54 (2)^***^
lung disease	166 (2.8)	20 (1.0)	43 (2.2)	103 (4.9)	58.63 (2)^***^
liver disease	165 (2.8)	39 (2.0)	69 (3.6)	57 (2.7)	9.03 (2)^*^
heart disease	593 (9.9)	75 (3.9)	168 (8.7)	350 (16.6)	187.67 (2)^***^
cerebrovascular disease	348 (5.8)	36 (1.9)	114 (5.9)	198 (9.4)	104.60 (2)^***^
psychiatric disease	294 (4.9)	40 (2.1)	112 (5.8)	142 (6.7)	52.09 (2)^***^
rheumatoid arthritis	1529 (25.6)	172 (8.9)	457 (23.8)	900 (42.7)	611.59 (2)^***^
digestive disorder	95 (1.6)	20 (1.0)	30 (1.6)	45 (2.1)	7.88 (2)^*^
Number of chronic diseases	1.3 (1.2)	0.6 (0.7)	1.3 (1.2)	1.9 (1.2)	742.75 (2, 3892)^***^
Alcohol use					388.05 (4)^***^
never	2899 (48.5)	788 (40.6)	889 (46.2)	1222 (57.9)	
former	1224 (20.5)	288 (14.8)	394 (20.5)	542 (25.7)	
current	1852 (31.0)	866 (44.6)	640 (33.3)	346 (16.4)	
Smoking					98.11 (4)^***^
never	4132 (69.2)	1355 (69.8)	1264 (65.7)	1513 (71.7)	
former	1371 (22.9)	376 (19.3)	475 (24.7)	520 (24.6)	
current	472 (7.9)	211 (10.9)	184 (9.6)	77 (3.7)	
Depressive symptom	1.3 (1.8)	1.1 (1.6)	1.2 (1.7)	1.7 (2.1)	49.13 (2, 3973.6)^***^
Self-rated health status	3.0 (0.8)	3.4 (0.7)	3.1 (0.8)	2.6 (0.8)	530.11 (2, 3972.3)^***^
Number of functional teeth	22.8 (9.5)	27.0 (4.8)	24.6 (7.6)	17.3 (11.5)	637.36 (2, 3642)^***^
Masticatory ability	3.2 (0.8)	3.6 (0.7)	3.2 (0.8)	2.7 (0.8)	665.40 (2, 3974)^***^
GOHAI	35.9 (7.0)	38.2 (5.9)	36.5 (6.2)	33.3 (7.8)	250.98 (2, 3965.8)^***^
K-MMSE	25.1 (5.4)	27.8 (2.8)	26.2 (4.0)	21.7 (6.5)	802.36 (2, 3718.5)^***^

^*^p < .05, ^**^p < .01, ^***^p < .001

### Correlation between oral health, general health, and cognitive function

Correlation analysis between oral health and factors influencing the level of cognitive function in the elderly has represented that oral health had a stronger correlation with sociodemographic characteristics and self-rated health status than with objective health status. Concerning sociodemographic characteristics, oral health was negatively correlated with age, *r* = −.47 – −.31, while positively correlated with educational level, *r* = .25 – .35, and with residence area, *r* = .03 – .06. The result also showed that oral health had a relation with sex, *r* = −.03, and marital status, *r* = .18 – .24. When it comes to self-rated health status, self-rated health status was most positively correlated with masticatory ability, *r* = .45, and depressive symptom was most negatively correlated with the GOHAI score, *r* = −.28, of all oral health variables. Oral health had a negative correlation with the number of chronic diseases, *r* = −.30 – −.22 and had a positive correlation with alcohol use, *r* = .09 – .12. Of the three oral health variables, masticatory ability showed the strongest correlation with cognitive function, *r* = .42, while GOHAI score did weakest correlation with it, *r* = .34. The result of the matrix of correlation analysis is summarized in < Table S1>.

### Hierarchical multiple linear regression results predicting cognitive function

A hierarchical multiple linear regression was implemented to test the predictions of cognitive function with four blocks of variables. By adding four blocks, each of which were oral health variables, sociodemographic variables, objective health status variables, and self-rated health status, as predictors in four regression models one at a time, changes of variances in the level of cognition were calculated to explore the different effect size of predictions by four models.

Of all statistical assumption testing on regression analysis, homoscedasticity and normality were slightly violated for the skewness of cognitive function; however, the heteroscedasticity issue was offset by additional subgroup analysis by age group and the sample size analysed in the study was large enough, being robust to violation of the normality assumption, to exclude the normality test [50]. Additionally, no multicollinearity was confirmed for any variables by the variance inflation factors (VIF), not exceeding 5 [51], then it gave this study a plausible reason for continuing regression analysis.

The results showed that all four models were statistically significant in predicting cognitive function. The first model itself accounted for 23% of the variance of cognitive function, *F*(3, 5971) = 609.50, *p* < .001, *R*^2^ = 0.23, and the second model explained 16% more of the variance when sociodemographic variables were included, *F*(14, 5960) = 270.50, *p* < .001, *R*^2^ = 0.39. The third model including objective health status represented little amount of but still significant 1% improvement of the variance, *F*(28, 5946) = 143.00, *p* < .001, *R*^2^ = 0.40. Final model with all variables increased by 7% of the variance from the third model, *F*(30, 5944) = 173.30, *p* < .001, *R*^2^ = 0.47. From the first model to final model, whether adjustment for other variables was applied to or not, three oral health condition remained the statistically significant predictors. Regarding on result from the fourth model in < Table 2>, cognitive function was positively associated with the number of functional teeth, masticatory ability, the GOHAI, education level, the residence area, diagnosis with diabetes mellitus, and with cancer and malignant tumours, and self-self-rated health status. On the other way, cognitive function was negatively associated with age, the number of chronic diseases, diagnosis with cerebrovascular disease, and rheumatoid arthritis, and the level of depressive symptoms. Sex and former alcohol use or smoking experience were related to the cognitive function of the elderly but marital status, diagnosis with hypertension, lung disease, liver disease, heart disease, digestive disorders, and psychiatric diseases were not.

**Table 2:** Result of hierarchical regression analysis

	Model 1			Model 2			Model 3			Model 4			
	*B*	*SE*	*t*	*B*	*SE*	*t*	*B*	*SE*	*t*	*B*	*SE*	*t*	VIF
Number of functional teeth	0.13	0.01	17.35^***^	0.05	0.01	6.55^***^	0.04	0.01	6.15^***^	0.04	0.01	5.63^***^	0.69
Masticatory ability	1.54	0.09	17.09^***^	0.75	0.08	8.96^***^	0.70	0.08	8.46^***^	0.39	0.08	4.86^***^	0.57
GOHAI	0.11	0.01	11.26^***^	0.08	0.01	9.13^***^	0.08	0.01	8.81^***^	0.03	0.01	3.85^***^	0.70
Age				-0.18	0.01	-22.22^***^	-0.16	0.01	-19.04^***^	-0.15	0.01	-19.12^***^	0.46
Sex (ref = male)				-0.75	0.13	-5.99^***^	-0.83	0.18	-4.69^***^	-0.84	0.17	-5.07^***^	0.39
Education level (ref = elementary)													0.57
middle school				1.73	0.17	10.03^***^	1.66	0.17	9.70^***^	1.49	0.16	9.15^***^	
high school				1.88	0.16	11.79^***^	1.73	0.16	10.83^***^	1.51	0.15	9.95^***^	
college				2.04	0.21	9.76^***^	1.84	0.21	8.79^***^	1.64	0.20	8.24^***^	
Marital status (ref = unmarried)													0.67
widowed				0.57	0.62	0.92	0.53	0.61	0.86	0.43	0.58	0.75	
divorced				0.80	0.69	1.15	0.91	0.69	1.33	1.05	0.65	1.62	
living apart together				1.23	0.96	1.28	1.47	0.95	1.55	1.05	0.90	1.17	
married				1.16	0.6	1.93	1.16	0.6	1.94	0.99	0.56	1.76	
Area of residence (ref = township)													0.91
small city				0.03	0.15	0.22	0.09	0.15	0.57	0.34	0.14	2.41^*^	
big city				0.54	0.15	3.67^***^	0.58	0.15	3.98^***^	0.53	0.14	3.88^***^	
Chronic disease													
hypertension							-0.07	0.12	-0.58	0.06	0.11	0.49	0.82
diabetes mellitus							0.17	0.14	1.19	0.28	0.13	2.11^*^	0.89
cancer and malignant tumours							0.19	0.21	0.92	0.63	0.20	3.21^**^	0.96
lung disease							-0.28	0.33	-0.83	-0.27	0.32	-0.86	0.98
liver disease							-0.35	0.33	-1.05	-0.33	0.32	-1.03	0.98
heart disease							-0.15	0.19	-0.81	0.05	0.18	0.30	0.94
cerebrovascular disease							-1.93	0.24	-8.10^***^	-1.30	0.23	-5.73^***^	0.93
psychiatric disease							-0.37	0.26	-1.44	0.25	0.24	1.03	0.95
rheumatoid arthritis							-0.64	0.14	-4.49^***^	-0.36	0.13	-2.71^**^	0.77
digestive disorder							0.45	0.44	1.03	0.76	0.41	1.85	0.99
Alcohol use (ref = never)													0.62
former							-0.48	0.16	-3.02^**^	-0.35	0.15	-2.34^*^	
current							0.31	0.15	2.04^*^	0.18	0.14	1.26	
Smoking (ref = never)													0.48
former							-0.31	0.18	-1.77	-0.35	0.17	-2.13^*^	
current							-0.25	0.24	-1.05	-0.39	0.23	-1.70	
Depressive symptom										-0.66	0.03	-21.88^***^	0.87
Self-rated health status										1.01	0.08	13.12^***^	0.65
*F*	609.50 (3, 5971)***			270.50 (14, 5960)***			143.00 (28, 5946)***			173.30 (30, 5944)***			
*R* ^2^	0.23			0.39			0.40			0.47			
*R*^2^ adjusted	0.23			0.39			0.40			0.46			
Δ*R*^2^				0.16			0.01			0.07			

^*^p < .05, ^**^p < .01, ^***^p < .001

Different types of variables have been accumulated as predictors from model 1 to model 4 as below

ㆍModel 1: 3 oral health variables

ㆍModel 2: 3 oral health variables + 5 sociodemographic variables

ㆍModel 3: 3 oral health variables + 5 sociodemographic variables + 3 objective health status variables

ㆍModel 4: 3 oral health variables + 5 sociodemographic variables + 3 objective health status variables + 2 subjective health status variables

Oral health variables: # of functional teeth, masticatory ability, GOHAI (Geriatric Oral Health Assessment Index)

Sociodemographic variables: age, sex, education level, marital status, area of residence

Systemic health variables: objective health status variables (diagnosed of 10 chronic diseases, alcohol use, smoking habit) + subjective health status variables (depressive symptoms, self-rated health status)

### Cognitive function by three age groups

Subgroup hierarchical multiple linear regression analyses by three age groups for cognitive function prediction were delivered in this study and the results were suggested in < Table 3> (for detailed results by each group, see < Table S2-S4>).

**Table 3:** Hierarchical Regression Analysis Result by Age Group

		< 65			65–74			≥ 75	
	*B*	*SE*	*t*	*B*	*SE*	*t*	*B*	*SE*	*t*
Number of functional teeth	< 0.001	0.01	-0.01	0.00	0.01	-0.17	0.04	0.01	3.62^***^
Masticatory ability	0.41	0.10	4.21^***^	0.39	0.13	3.07^**^	0.60	0.16	3.68^***^
GOHAI	0.03	0.01	2.65^**^	0.03	0.02	2.36^*^	0.02	0.02	1.29
Age	-0.01	0.03	-0.53	-0.14	0.03	-4.71^***^	-0.32	0.03	-12.66^***^
Sex (ref = male)	-0.05	0.18	-0.27	-0.41	0.26	-1.55	-1.75	0.39	-4.49^***^
Education level (ref = elementary)									
middle school	0.90	0.24	3.75^***^	1.19	0.22	5.33^***^	1.50	0.35	4.26^***^
high school	1.39	0.21	6.58^***^	1.43	0.22	6.60^***^	1.86	0.34	5.43^***^
college	1.64	0.24	6.75^***^	1.71	0.30	5.62^***^	2.61	0.50	5.20^***^
Marital status (ref = unmarried)									
widowed	1.24	0.53	2.35^*^	-0.05	0.96	-0.06	-1.21	2.29	-0.53
divorced	1.75	0.54	3.23^**^	-0.40	1.06	-0.38	-0.84	2.48	-0.34
living apart together	0.32	0.84	0.38	0.93	1.32	0.71	-0.82	3.22	-0.25
married	1.42	0.46	3.09^**^	0.07	0.94	0.07	-1.16	2.29	-0.51
Residence (ref = township)									
small city	0.00	0.18	-0.01	0.44	0.22	2.02^*^	0.50	0.29	1.74
big city	0.33	0.17	1.914.	0.81	0.21	3.88^***^	0.55	0.28	1.99^*^
Chronic disease									
hypertension	0.06	0.14	0.39	-0.20	0.17	-1.18	-0.08	0.24	-0.33
diabetes mellitus	0.15	0.20	0.77	0.15	0.20	0.78	0.17	0.25	0.65
cancer and malignant tumours	0.14	0.27	0.52	0.33	0.29	1.15	0.61	0.39	1.58
lung disease	0.09	0.58	0.15	0.16	0.54	0.29	-0.49	0.52	-0.94
liver disease	0.27	0.42	0.64	-0.30	0.43	-0.70	-1.06	0.68	-1.54
heart disease	-0.38	0.31	-1.23	0.04	0.29	0.16	0.12	0.30	0.41
cerebrovascular disease	-0.77	0.44	-1.74	-1.29	0.35	-3.72^***^	-1.52	0.39	-3.92^***^
psychiatric disease	-0.23	0.42	-0.54	0.10	0.35	0.29	0.08	0.45	0.19
rheumatoid arthritis	-0.40	0.21	-1.86	-0.48	0.20	-2.36^*^	-0.20	0.25	-0.79
digestive disorder	0.51	0.58	0.89	0.84	0.64	1.31	0.72	0.77	0.94
Alcohol use (ref = never)									
former	-0.47	0.18	-2.58^**^	-0.42	0.23	-1.81	-0.40	0.31	-1.26
current	0.10	0.14	0.68	0.19	0.21	0.91	0.39	0.37	1.06
Smoking (ref = never)									
former	-0.16	0.19	-0.83	-0.13	0.26	-0.52	-0.75	0.34	-2.20^*^
current	0.01	0.23	0.03	0.07	0.33	0.20	-0.90	0.64	-1.41
Depressive symptom	-0.34	0.04	-8.91^***^	-0.62	0.05	-12.70^***^	-0.81	0.06	-14.10^***^
Self-rated health status	0.48	0.10	5.01^***^	0.85	0.12	7.17^***^	1.34	0.16	8.66^***^
*F*	15.14 (30, 1911)^***^			24.32 (30, 1892)^***^			45.64 (30, 2079)^***^		
*R* ^2^	0.19			0.28			0.40		
*R*^2^ adjusted	0.18			0.27			0.39		

^*^p < .05, ^**^p < .01, ^***^p < .001

The number of functional teeth had a positive relation to cognitive function in those aged over 75 years, *B* = 0.04, *p* < .001. The more masticatory ability people had, the better cognitive function they retained in all age groups: 45–64 years, *B* = 0.41, *p* < .001, 65–74 years, *B* = 0.39, *p* = .002, and over 74 years, *B* = 0.60, *p* < .001. The high GOHAI score predicted the increased level of cognitive function for those under 65 years of age, *B* = 0.03, *p* = .008 and those 65–74 years of age, *B* = 0.03, *p* = .019.

Age was found to be a statistically significant predictor of cognitive function both in people aged 65–74 years, *B* = −0.14, *p* < .001, and 75 and older, *B* = −0.32, *p* < .001. In terms of sex, males over 74 years of age had better cognitive function than females, *B* = −1.75, *p* < .001. The education level had a positive association with cognitive function in all age groups, and marital status had a significant association with cognitive function for those under 65 years of age. People living in a large city compared to a township among those aged 65–74 years, *B* = 0.81, *p* < .001, and over 74 years, *B* = 0.55, *p* = .047, had better cognitive function.

Having been diagnosed with cerebrovascular disease both in the group of 65–74 years of age, and those over 74 years of age had significantly lower cognitive function, *B* = −1.29, *p* < .001, and, *B* = −1.52, *p* < .001, respectively, than those who have not been with; and people having had rheumatoid arthritis only in the group of aged 65–74 years showed decreased level of cognitive function, *B* = −0.48, *p* = .018. Concerning alcohol drinking or smoking habits, the elderly who had experienced but were not currently drinking under the age of 65 and former smokers over the age of 74 both have shown declines in cognitive function, *B* = −0.47, *p* = .010. *B* = −0.75, *p* = .028.

The degree of depressive symptoms had a negative association with cognitive function in all age groups: under 65 years, *B* = −0.34, *p* < .001; 65–74 years, *B* = −0.62, *p* < .001; and over 74 years, *B* = −0.81, *p* < .001. The association between the self-rated health status and the level of cognitive function has also been verified in all age groups: under 65 years, *B* = 0.48, *p* < .001, 65–74 years old, *B* = 0.85, *p* < .001, and over 74 years, *B* = 1.34, *p* < .001.

## Discussion

This study aimed to confirm the association of oral health and systemic health on cognitive function in people aged 55 and over and to investigate the differences by age group using the latest cross-sectional dataset from a biannual longitudinal panel survey in Korea. Oral health accounted for 23.4% of cognitive function and cognitive function was positively correlated with the number of functional teeth, masticatory ability, and the GOHAI score. Notably, when controlling for sociodemographic and systemic health variables, masticatory ability was found to be a significant factor in explaining cognitive function in all age groups aged over 54. However, significant associations between cognitive function and the GOHAI scores were observed in the 55–74 age group, while the number of functional teeth showed significance in the age group aged > 74 years. These results support previous studies showing that oral diseases such as periodontitis [15–17], the number of teeth [18–20] and chewing efficiency [21–23] are associated with cognitive ability in the elderly population. The age-dependency of the oral variables necessitates the implementation of age-group-specific policy-driven systematic oral healthcare services along with proper education programs to ease the socioeconomic and medical burden originating from cognitive decline in older adults.

Oral variables correlated with the number of chronic diseases and the diagnosis of specific chronic diseases, including hypertension, diabetes mellitus, chronic lung disease, heart disease, cerebrovascular disease, psychiatric disease, and rheumatoid arthritis. By contrast, no correlations were found with cancer or malignant tumours, liver disease, or digestive disease. Regarding health-related behaviours, alcohol use experience but not smoking was found to be correlated with oral health status. These results were partially supported by previous studies reporting that dental conditions such as periodontitis, tooth loss, and caries have correlations with diabetes mellitus, cardiovascular disease, and cerebrovascular disease [52, 53]. Some of the findings, however, are inconsistent with previous studies showing that periodontal disease including tooth loss is associated with an increased risk of cancers [54, 55] and that smoking increases the risk of tooth loss and edentulism [56, 57].

The discoveries on the positive association between cognitive function with diabetes or cancer in this study deviate from conventional findings – diabetes predicting increased incidence of cognition loss or abnormality [58, 59] and commonly reported cancer-related cognitive impairment [60–62]. However, recent studies have reported similar results to those found in our research. One retrospective study with amnestic cognitive impairment subjects in Chile and other population-based US cohort study with 14,583 participants conducted from 1998 to 2014 have shown epidemiological negative associations between cancer and cognitive decline. These two studies supported the hypothesis of a biological inverse mechanism between carcinogenesis and neurodegeneration [63, 64]. Apart from the biological explanation, we posit that the findings could be ascribed to the management of a healthy lifestyle. The concept of a healthy lifestyle perspective may align with the previous literature exploring the association of sustaining physical activity [65–67], high-quality sleep [68–71], a healthy dietary pattern adherence with [72, 73] cognitive decline attenuation or cognitive function enhancement with cancer or diabetes. When delving into the relationships encompassing oral health, systemic health, and cognition, it would be essential to adopt a multifaceted approach and to incorporate variables of healthy lifestyle and behaviours into the in-depth interpretation.

Depression and self-rated health status also appear to have a significant correlation with oral health, supporting previous studies that oral health is inversely related to depression [74–76]. The significant correlation between masticatory ability and self-rated health status is self-explanatory in that masticatory movement is important for eating enjoyment without limiting the variety of food and nutrition supply [77]. Thus, it is suggested that for proper management of depression in the elderly group, a holistic healthcare program comprising not only psychological care but also oral care with proper diet and lifestyle is needed.

Previously, cognitive functions have been reported to be lower in people with lower socioeconomic status (SES), increasing age, and in women [78–82]. This study also showed that cognitive function declined more severely in women, less-educated people, and people not living in a big city, with age being the most influential variable. Notably, a recent analysis of two UK-based prospective cohort studies, including 15,924 participants from the English Longitudinal Study of Ageing (ELSA) and the Whitehall II study, revealed that disparities between sexes in education yielded sex differences in cognitive outcomes in older adults [83]. The seminal study also found that women, with evidence of a slower memory decline, have higher fluency scores than men in the high-education group. Thus, the sex differences in cognitive functions in the current study should be interpreted with social conditions in which the degree of inequality in education increases with age. It is also supported by the literature indicating the connection between early SES and a healthy lifestyle mediated by educational attainment, which in turn predicts cognitive abilities in later life stages [84–86]. Not reported as main findings in this study, other SES variables such as income or assets exhibited a significant association with oral health as well as cognitive function. Previous studies explained the impact of childhood family income on brain development and cognitive performance and the association between wealth and adverse alterations in physical, social, emotional, and cognitive levels in later stages of life [87, 88]. Therefore, economic status should be additionally considered as another significant factor to explain cognitive function in the elderly.

This study suggested evidence showing the association between oral health, systemic health, and cognitive function comprehensively and additional age-specific characteristics in predicting cognitive function. There were several limitations to be discussed for designing further study. First, the data collection procedure for panel data might raise potential concerns regarding measurement error. This panel data was gathered based on a self-reported survey without the aid of objective examination or professional medical staff. The accuracy of diagnosis information relied on the respondents’ memory, and the quantification of the number of teeth was based on self-counting rather than test-based measurement, all of which could lead to less precise data collection. Like other surveys using the CAPI system, interviewer bias or interviewer-respondent interaction bias may not have been eliminated. Even though all investigators had been well-trained by KEIS before the survey, it might have been challenging to secure the same investigators or equally qualified ones in every wave of the survey. This could potentially influence the responses obtained from participants and eventually affect the accuracy of the result.

Second, this research may encounter biased estimation and interpretation issues by the operationalization of the definition and analytical techniques. We primarily defined functional teeth based on previous literature [89, 90], emphasizing their role in mastication capability. It has been finalized the concept of functional teeth after conducting a correlation analysis between several possible combinations with five types of ‘the number of teeth’ variables collectively in the given dataset and cognitive function – selecting the one from the highest correlation coefficient value. Both considering theory-driven and data-driven operationalization could establish more robust and generalizable findings; however, there remained instability, subjectivity, and difficulties in reproducibility or generalization issues caused by data-oriented processing.

Another issue that may hamper the interpretation of the results pertains to the use of an unweighted regression analysis. In the context of survey data, not considering the complexities of the sampling design would result in a biased estimation of the coefficient affecting statistical significance tests and misleading conclusions. The last issue which requires cautious interpretation is the analytical technique used in this study. Despite having access to panel data that could track individuals over time, the analysis was limited to the use of cross-sectional data. It has not been verified that the causality between predictors – oral health, systemic health, sociodemographic characteristics – and cognitive function, and the identification of moderating or mediating effects among the study variables explaining cognitive function.

Taking the limitations affecting the reliability and validity of study results into account for future studies, it is necessary to employ a longitudinal analysis approach for investigating the directionality of the effects among oral, systemic, and cognitive health variables. It is also possible to design further research to explore the intricate association between oral or systemic health status and cognitive functioning while considering the lifestyle factors and SES for suggesting ground evidence on healthy late-life experience.

## Conclusions

This study conducted an integrated analysis of the relationship between oral health, systemic health, and cognitive function in the elderly population. Hierarchical analysis results revealed the relatively prominent importance of oral health for cognitive function among individuals above the age of 54. Each oral variable such as the number of functional teeth, masticatory ability, and GOHAI, however, differently represented the association on cognitive function by the pre-, the early-, and the middle-to-late-old groups: masticatory ability commonly related to cognitive ability in all groups, self-perceived oral health in the pre- and the early elderly groups, and the number of functional teeth in the middle-to-late elderly group.

Regarding the relationship between systemic health status and elderly cognitive function, positive health perceptions were associated with better cognitive ability in all groups, but in the early elderly, cognitive decline was assocated with cerebrovascular disease or rheumatoid arthritis. This study confirmed the strong evidence of educational level relating to cognitive function in elderly individuals. It also revealed that middle-to-late elderly females exhibited a lower level of cognitive function compared to males of the same age group. Furthermore, age and residence area size seemed to have a significant association with cognitive function from the onset of the early elderly period.

Importantly, this study provides essential groundwork for establishing a holistic approach to cognition healthcare policy tailored to the elderly population while considering different age groups and various factors including oral health, systemic health, and sociodemographic features.

### Electronic supplementary material

Below is the link to the electronic supplementary material.

Supplementary Material 1

## Data Availability

The KLoSA data with deleted personal information and the detailed survey design are publicly available after member registration at https://survey.keis.or.kr/eng/myinfo/login.jsp. Gateway to Global Aging site (https://g2aging.org/?section=study&studyid=5) also contains significant information on KLoSA including codebooks, harmonizing code and other useful resources.
