# Supplementary material for: Age-group-specific association of oral health and systemic health on cognitive function: a cross-sectional study of Korean elders
Source: BMC Oral Health. 2023 Dec 13;23:997. doi: 10.1186/s12903-023-03724-2 (PMC10720108; doi:10.1186/s12903-023-03724-2)
Supplement: Supplementary file 1 — Supplementary Material 1 [file 12903_2023_3724_MOESM1_ESM.pdf]

Supplementary Information containing Tables S1-S4.

Age-group-specific association of oral health and systemic health on cognitive function: a cross-sectional study of Korean elders

Jae-Eun Sin[1], Hye-Sung Kim[1,2], Inseong Hwang[3], Miwha Noh[2]

[1] Apple Tree Institute of Biomedical Science, Apple Tree Medical Foundation, 1450 Jungang-ro, Goyang-si, Gyeonggi-do, Republic of Korea

[2] Apple Tree Dental Hospital, Apple Tree Medical Foundation, 1450 Jungang-ro, Goyang-si, Gyeonggi-do, Republic of Korea

[3] DOCSmedi OralBiome Co., Ltd., 143 Gangseong-ro, Goyang-si, Gyeonggi-do, Republic of Korea

Correspondence

Inseong Hwang, DOCSmedi OralBiome Co., Ltd., 143 Gangseong-ro, Goyang-si, Gyeonggi-do, Republic of Korea.

Email: inseong.hwang@gmail.com

Miwha Noh, Apple Tree Medical Foundation, 1450 Jungang-ro, Goyang-si, Gyeonggi-do, Republic of Korea

Email: miwha6708@naver.com

<Table S1> Result of the correlation analysis between oral health, general health, and cognitive function.

	Number of functional teeth	Masticatory ability	GOHAI
Age	- .47***	- .45***	- .31***
Sex	- .03*	- .03*	- .03**
Education level	.35***	.33***	.25***
Marital status	.24***	.21***	.18***
Residence	.06***	.08***	.03**
Hypertension	- .20***	- .20***	- .15***
Diabetes mellitus	- .14***	- .16***	- .13***
Cancer and malignant tumours	n.s.	- .04**	n.s.
Lung disease	- .07***	- .05***	- .05***
Liver disease	n.s.	n.s.	n.s.
Heart disease	- .10***	- .11***	- .09***
Cerebrovascular disease	- .10***	- .12***	- .08***
Psychiatric disease	- .07***	- .07***	- .07***
Rheumatoid arthritis	- .22***	- .22***	- .16***
Digestive disorder	n.s.	- .05***	n.s.
Number of chronic diseases	- .25***	- .30***	- .22***
Alcohol use	.12***	.12***	.09***
Smoking	n.s.	n.s.	n.s.
Depressive symptom	- .15***	- .20***	- .28***
Self-rated health status	.30***	.45***	.30***
Cognitive function	.38***	.42***	.34***

$^{*}p<.05$, $^{**}p<.01$, $^{***}p<.001$

<Table S2> Result of Hierarchical Regression Analysis of Age Group (<65).

	Model 1			Model 2			Model 3			Model 4			
	B	*SE*	*t*	*B*	*SE*	*t*	*B*	*SE*	*t*	*B*	*SE*	*t*	VIF
Number of functional teeth	0.01	0.01	0.95	0.00	0.01	0.06	0.00	0.01	-0.06	0.00	0.01	-0.01	1.10
Masticatory ability	0.76	0.10	7.77***	0.63	0.10	6.61***	0.63	0.10	6.54***	0.41	0.10	4.21***	1.41
GOHAI	0.06	0.01	4.87***	0.05	0.01	4.57***	0.05	0.01	4.32***	0.03	0.01	2.65**	1.31
Age				-0.03	0.03	-1.02	-0.01	0.03	-0.32	-0.01	0.03	-0.53	1.18
Sex (ref = male)				-0.10	0.13	-0.77	-0.05	0.18	-0.26	-0.05	0.18	-0.27	2.28
Education level (ref = elementary)													1.38
middle school				0.96	0.24	3.90***	0.90	0.25	3.68***	0.90	0.24	3.75***	
high school				1.57	0.22	7.28***	1.43	0.22	6.60***	1.39	0.21	6.58***	
college				1.82	0.25	7.36***	1.67	0.25	6.71***	1.64	0.24	6.75***	
Marital status (ref = unmarried)													1.15
widowed				1.34	0.54	2.47*	1.41	0.54	2.61**	1.24	0.53	2.35*	
divorced				1.69	0.56	3.02**	1.72	0.56	3.08**	1.75	0.54	3.23**	
living apart together				0.45	0.87	0.52	0.57	0.87	0.65	0.32	0.84	0.38	
married				1.54	0.47	3.26**	1.58	0.47	3.34***	1.42	0.46	3.09**	
Area of residence (ref = township)													1.14
small city				-0.09	0.18	-0.52	-0.07	0.18	-0.40	0.00	0.18	-0.01	
big city				0.42	0.18	2.38*	0.42	0.18	2.38*	0.33	0.17	1.91	
Chronic disease													
hypertension							-0.02	0.14	-0.15	0.05	0.14	0.39	1.12
diabetes mellitus							0.08	0.20	0.42	0.15	0.20	0.77	1.09
cancer or malignant tumours							-0.06	0.28	-0.23	0.14	0.27	0.52	1.06
lung disease							-0.01	0.59	-0.01	0.08	0.58	0.15	1.02
liver disease							0.20	0.43	0.47	0.27	0.42	0.64	1.03
heart disease							-0.53	0.31	-1.69	-0.38	0.31	-1.23	1.04
cerebrovascular disease							-1.26	0.45	-2.79**	-0.77	0.44	-1.74	1.08
psychiatric disease							-0.54	0.43	-1.26	-0.23	0.42	-0.54	1.06
rheumatoid arthritis							-0.50	0.22	-2.28*	-0.40	0.21	-1.86	1.11
digestive disorder							0.34	0.59	0.58	0.51	0.58	0.89	1.02
Alcohol use (ref = never)													1.38
former							-0.55	0.19	-2.92**	-0.47	0.18	-2.58**	
current							0.10	0.15	0.65	0.10	0.14	0.68	
Smoking (ref = never)													2.11
former							-0.10	0.20	-0.49	-0.16	0.19	-0.83	
current							0.10	0.24	0.43	0.01	0.23	0.03	
Depressive symptom										-0.34	0.04	-8.91***	1.13
Self-rated health status										0.48	0.09	5.01***	1.29
F	49.55 (3, 1938)***			20.30 (14, 1927)***			11.63 (28, 1913)***			15.14 (30, 1911)***			
R^2	.07			.13			.15			.19			
R^2 adjusted	.07			.12			.13			.18			
ΔR^2				.06			.02			.05			

* p<.05, ** p<.01, *** p<.001

<Table S3> Result of Hierarchical Regression Analysis of Age Group (65-74).

	Model 1			Model 2			Model 3			Model 4			
	B	*SE*	*t*	*B*	*SE*	*t*	*B*	*SE*	*t*	*B*	*SE*	*t*	VIF
Number of functional teeth	0.03	0.01	2.38*	0.01	0.01	0.63	0.01	0.01	0.47	0.00	0.01	-0.17	1.25
Masticatory ability	0.87	0.14	6.41***	0.69	0.13	5.28***	0.62	0.13	4.74***	0.39	0.13	3.07**	1.49
GOHAI	0.08	0.02	5.15***	0.08	0.02	5.08***	0.07	0.02	4.96***	0.03	0.01	2.36*	1.31
Age				-0.15	0.03	-4.95***	-0.12	0.03	-4.07***	-0.14	0.03	-4.71***	1.13
Sex (ref = male)				-0.39	0.19	-2.11*	-0.29	0.28	-1.04	-0.41	0.26	-1.55	2.75
Education level (ref = elementary)													1.38
middle school				1.57	0.24	6.66***	1.40	0.24	5.94***	1.19	0.22	5.33***	
high school				1.95	0.23	8.60***	1.73	0.23	7.58***	1.43	0.22	6.60***	
college				2.27	0.32	7.11***	1.95	0.32	6.06***	1.71	0.30	5.62***	
Marital status (ref = unmarried)													1.23
widowed				0.03	1.02	0.03	0.01	1.02	0.01	-0.05	0.96	-0.06	
divorced				-0.49	1.13	-0.43	-0.39	1.12	-0.35	-0.40	1.06	-0.38	
living apart together				1.06	1.40	0.76	1.26	1.40	0.9	0.93	1.32	0.71	
married				0.22	1.00	0.22	0.19	1.00	0.19	0.07	0.94	0.07	
Area of residence (ref = township)													1.08
small city				0.09	0.23	0.39	0.17	0.23	0.76	0.44	0.22	2.02*	
big city				0.65	0.22	2.90**	0.75	0.22	3.38***	0.81	0.21	3.88***	
Chronic disease													
hypertension							-0.34	0.18	-1.94	-0.20	0.17	-1.18	1.12
diabetes mellitus							0.04	0.21	0.2	0.15	0.20	0.78	1.10
cancer or malignant tumours							-0.09	0.30	-0.31	0.33	0.29	1.15	1.05
lung disease							-0.24	0.57	-0.43	0.16	0.54	0.29	1.03
liver disease							-0.29	0.45	-0.65	-0.30	0.43	-0.7	1.02
heart disease							-0.04	0.30	-0.15	0.04	0.29	0.16	1.05
cerebrovascular disease							-1.85	0.36	-5.10***	-1.29	0.35	-3.72***	1.08
psychiatric disease							-0.52	0.37	-1.4	0.10	0.35	0.29	1.10
rheumatoid arthritis							-0.68	0.21	-3.18**	-0.48	0.20	-2.36*	1.21
digestive disorder							0.67	0.68	0.99	0.84	0.64	1.31	1.01
Alcohol use (ref = never)													1.58
former							-0.41	0.25	-1.69	-0.42	0.23	-1.81	
current							0.29	0.23	1.3	0.19	0.21	0.91	
Smoking (ref = never)													2.26
former							-0.16	0.27	-0.6	-0.13	0.26	-0.52	
current							0.19	0.35	0.54	0.07	0.33	0.2	
Depressive symptom										-0.62	0.05	-12.70***	1.12
Self-rated health status										0.85	0.12	7.17***	1.31
F	51.56 (3, 1919)***			26.36 (14, 1908)***			15.98 (28, 1894)***			24.32 (30, 1892)***			
R^2	.07			.16			.19			.28			
R^2 adjusted	.07			.16			.18			.27			
ΔR^2				.09			.03			.09			

*p<.05, **p<.01, ***p<.001

<Table S4> Result of Hierarchical Regression Analysis of Age Group (≥75).

	Model 1			Model 2			Model 3			Model 4			
	B	*SE*	*t*	*B*	*SE*	*t*	*B*	*SE*	*t*	*B*	*SE*	*t*	VIF
Number of functional teeth	0.09	0.01	7.07***	0.05	0.01	4.34***	0.05	0.01	3.98***	0.04	0.01	3.62***	1.23
Masticatory ability	1.40	0.19	7.48***	0.99	0.17	5.71***	0.91	0.17	5.29***	0.60	0.16	3.68***	1.50
GOHAI	0.13	0.02	6.74***	0.09	0.02	5.38***	0.08	0.02	4.99***	0.02	0.02	1.29	1.35
Age				-0.37	0.03	-13.86***	-0.35	0.03	-13.30***	-0.32	0.02	-12.66***	1.27
Sex (ref = male)				-1.50	0.30	-4.95***	-1.76	0.42	-4.23***	-1.75	0.39	-4.49***	3.01
Education level (ref = elementary)													1.44
middle school				1.78	0.38	4.66***	1.71	0.38	4.54***	1.50	0.35	4.26***	
high school				2.27	0.37	6.16***	2.16	0.37	5.91***	1.86	0.34	5.43***	
college				3.21	0.54	6.00***	3.12	0.53	5.84***	2.61	0.50	5.20***	
Marital status (ref = unmarried)													1.58
widowed				-1.77	2.46	-0.72	-2.62	2.45	-1.07	-1.21	2.29	-0.53	
divorced				-2.26	2.67	-0.85	-2.67	2.66	-1.01	-0.84	2.48	-0.34	
living apart together				-2.00	3.47	-0.58	-1.99	3.45	-0.58	-0.82	3.22	-0.25	
married				-1.78	2.46	-0.72	-2.57	2.45	-1.05	-1.16	2.29	-0.51	
Area of residence (ref = township)													1.11
small city				0.00	0.31	0	0.14	0.31	0.45	0.50	0.29	1.74	
big city				0.48	0.30	1.59	0.58	0.30	1.95	0.55	0.28	1.99*	
Chronic disease													
hypertension							-0.18	0.26	-0.71	-0.08	0.24	-0.33	1.12
diabetes mellitus							0.03	0.27	0.11	0.16	0.25	0.65	1.10
cancer or malignant tumours							-0.01	0.41	-0.04	0.61	0.39	1.58	1.04
lung disease							-0.16	0.56	-0.29	-0.49	0.52	-0.94	1.04
liver disease							-0.97	0.73	-1.33	-1.06	0.68	-1.54	1.02
heart disease							-0.19	0.32	-0.58	0.12	0.30	0.41	1.05
cerebrovascular disease							-2.24	0.41	-5.44***	-1.52	0.39	-3.92***	1.06
psychiatric disease							-0.63	0.48	-1.33	0.08	0.45	0.19	1.04
rheumatoid arthritis							-0.63	0.26	-2.41*	-0.20	0.25	-0.79	1.24
digestive disorder							0.21	0.82	0.26	0.72	0.77	0.94	1.02
Alcohol use (ref = never)													1.81
former							-0.66	0.34	-1.98*	-0.40	0.31	-1.26	
current							0.92	0.40	2.33*	0.39	0.37	1.06	
Smoking (ref = never)													1.90
former							-0.70	0.36	-1.93	-0.75	0.34	-2.20*	
current							-0.68	0.68	-0.99	-0.90	0.64	-1.41	
Depressive symptom										-0.81	0.06	-12.70***	1.16
Self-rated health status										1.34	0.15	7.17***	1.36
F	110.10 (3, 2106)***			58.98 (14, 2095)***			32.92 (28, 2081)***			45.64 (30, 2079)***			
R^2	.14			.28			.31			.40			
R^2 adjusted	.13			.28			.30			.39			
ΔR^2				.15			.02			.09			

$^{*}p<.05$, $^{**}p<.01$, $^{***}p<.001$
